# Cost-effectiveness of blended vs. face-to-face cognitive behavioural therapy for severe anxiety disorders: study protocol of a randomized controlled trial

**DOI:** 10.1186/s12888-015-0697-1

**Published:** 2015-12-12

**Authors:** Geke Romijn, Heleen Riper, Robin Kok, Tara Donker, Maartje Goorden, Leona Hakkaart van Roijen, Lisa Kooistra, Anton van Balkom, Jeroen Koning

**Affiliations:** Faculty of Behavioural and Movement Sciences, Department of Clinical Psychology, VU University Amsterdam, Van der Boechorststraat 1, BT 1081 Amsterdam, The Netherlands; Psychiatric centre Pro Persona, Siependdaallaan 3, 4003 LE Tiel, The Netherlands; National Institute for Mental Health Research, The Australian National University, Building 63 Eggleston Road, Acton, ACT 2601 Australia; EMGO Institute for Health Care and Research, VU University Medical Centre, Van der Boechorststraat 7, BT 1081 Amsterdam, The Netherlands; Department of Psychiatry, VU University Medical Centre and GGZ inGeest, P.O. Box 7057, Amsterdam, MB 1007 The Netherlands; Institute for Medical Technology Assessment (iMTA), Erasmus University, PO box 1738, Rotterdam, The Netherlands; Telepsychiatry Unit, Southern Denmark University, Campusvej 55, DK 5230 Odense M, Denmark

**Keywords:** Anxiety disorders, Panic disorders, Generalized anxiety disorder, Social phobia, Internet-based treatment, Blended CBT, Cognitive behavioural therapy, Cost-effectiveness, Specialized mental health care, Randomized controlled trial

## Abstract

**Background:**

Anxiety disorders are among the most prevalent psychiatric conditions, and are associated with poor quality of life and substantial economic burden. Cognitive behavioural therapy is an effective treatment to reduce anxiety symptoms, but is also costly and labour intensive. Cost-effectiveness could possibly be improved by delivering cognitive behavioural therapy in a blended format, where face-to-face sessions are partially replaced by online sessions. The aim of this trial is to determine the cost-effectiveness of blended cognitive behavioural therapy for adults with anxiety disorders, i.e. panic disorder, social phobia or generalized anxiety disorder, in specialized mental health care settings compared to face-to-face cognitive behavioural therapy. In this paper, we present the study protocol. It is hypothesized that blended cognitive behavioural therapy for anxiety disorders is clinically as effective as face-to-face cognitive behavioural therapy, but that intervention costs may be reduced. We thus hypothesize that blended cognitive behavioural therapy is more cost-effective than face-to-face cognitive behavioural therapy.

**Methods/design:**

In a randomised controlled equivalence trial 156 patients will be included (*n* = 78 in blended cognitive behavioural therapy, *n* = 78 in face-to-face cognitive behavioural therapy) based on a power of 0.80, calculated by using a formula to estimate the power of a cost-effectiveness analysis: $n=\frac{2{\left({z}_a+{z}_{\beta}\right)}^2\left(s{d}^2+\left({W}^2s{d}^2\right)-\left(2W\rho s{d}_cs{d}_q\right)\right)}{{\left( WE-C\right)}^2}$. Measurements will take place at baseline, midway treatment (7 weeks), immediately after treatment (15 weeks) and 12-month follow-up. At baseline a diagnostic interview will be administered. Primary clinical outcomes are changes in anxiety symptom severity as measured with the Beck Anxiety Inventory. An incremental cost-effectiveness ratio will be calculated to obtain the costs per quality-adjusted life years (QALYs) measured by the EQ-5D (5-level version). Health-economic outcomes will be explored from a societal and health care perspective.

**Discussion:**

This trial will be one of the first to provide information on the cost-effectiveness of blended cognitive behavioural therapy for anxiety disorders in routine specialized mental health care settings, both from a societal and a health care perspective.

**Trial registration:**

Netherlands Trial Register NTR4912. Registered 13 November 2014.

## Background

Anxiety disorders are among the most prevalent psychiatric disorders worldwide [1]. They are associated with poor quality of life and a substantial economic burden [2–4].

Estimates of annual health care costs associated with anxiety disorders in the U.S. lie between $42 billion [5] and $47 billion [6]. A measure of overall disease burden is the disability-adjusted life year (DALY), expressed as the number of years lost due to ill health, disability or early death. The total global disease burden caused by anxiety disorders was 390 DALYs per 100,000 persons in 2010, being the sixth leading cause of disability [7].

In the Netherlands, annual health care costs are estimated at €286 million. In 2007, anxiety disorders accounted for 202,000 DALYs in the Netherlands, being third in the top ten list of medical disorders and having a higher cost impact than depression, diabetes mellitus or lung cancer [3].

Appropriate and efficient treatments are essential to reduce the impact of severe anxiety disorders on public health. These disorders can be treated effectively with cognitive-behavioural therapies (CBT), whether or not combined with pharmacotherapy. CBT is regarded as one of the preferred treatments for anxiety disorders in the Netherlands, set out in the multidisciplinary guidelines for anxiety [8] and international treatment guidelines [9, 10]. However, less than half of the patients with anxiety disorders receive appropriate treatment [11], due to anxiety-related avoidance behaviour, stigmatisation, waiting lists, costs of therapy and distance from service locations [12–14].

In recent years effort has been put in making less expensive and easily accessible interventions available for anxiety disorders while ensuring clinical effectiveness. These include self-help interventions. Studies indicate that these interventions did significantly better than waiting lists in terms of reducing anxiety symptoms [15]. Another important strategy for lowering treatment costs and improving accessibility are Internet interventions for mental disorders such as depression, anxiety disorders and problem drinking. Several meta-analyses have demonstrated that anxiety treatment delivered via Internet is more effective than non-intervening and that it can be as effective as face-to-face treatment [14–22]. Reger and Gahm [14], for example, showed that Internet- and computer-based treatments for anxiety disorders were superior to waitlist and that effects were equal to therapist-delivered treatment. Cuijpers et al. [18] investigated the effects of guided self-help on depression and anxiety compared to face-to-face psychotherapies and found no differences between the effects of both interventions. Andersson et al. [16] investigated the efficacy of guided Internet-based CBT (iCBT) in direct comparison to face-to-face CBT (fCBT) for psychiatric and somatic disorders. They concluded that both treatments produce equivalent effects.

Increasing emphasis is placed on cost-effectiveness of health care programmes, because of pressure on health care resources across the globe. In general, Internet interventions may be more cost-effective than face-to-face treatment. This has been confirmed in a recent systematic review by Donker et al. [23], in which 16 studies with economic evaluations of Internet interventions for anxiety, depression, smoking cessation and alcohol consumption were included. Nordgren et al. [24], for example, compared iCBT to an active waiting list control condition and found it to be cost-effective for primary-care patients with anxiety disorders with an ICER of − $1824, indicating lower costs and larger clinical effects in iCBT at post-test.

A rather new treatment approach combines face-to-face treatment with Internet components into one integrated treatment protocol [26]. This is called blended treatment [25]. Using this approach, part of the face-to-face treatment is replaced by Internet components, while the traditional face-to-face relationship between therapist and patient is retained. Blended treatment could possibly lower the number of face-to-face contacts, increase self-management competencies of patients and thereby reduce the overall (direct) treatment costs. This approach could also have a positive effect on waitlist periods, as it is expected that therapists can take on more patients, thereby reducing the number of patients that are waitlisted [26]. Therefore, blended treatments appear an attractive alternative for treatment as usual. However, little is known about the clinical outcome and cost-effectiveness of these treatments. In a recent study, Volker et al. [27] investigated the effectiveness of a blended intervention versus treatment as usual for sick-listed employees with common mental disorders, such as depression, anxiety and somatization disorders. Results demonstrated that the group receiving the blended intervention returned to work faster (27 days earlier on average) and had a greater chance of achieving remission than the group receiving treatment as usual. As far as we know there is no study yet investigating the cost-effectiveness of blended treatment for anxiety disorders.

We therefore aimed to investigate the cost-effectiveness of blended CBT (bCBT) for severe anxiety disorders. This refers to the group of patients with an anxiety disorder (panic disorder, social phobia or generalized anxiety disorder) who are referred to outpatient specialized mental health care. Treating these patients in primary care settings is not intensive enough given the severity of their disorders, for example due to pervasive avoidance behaviour leading to functional disabilities, or because of comorbidities that hamper treatment in primary care.

We hypothesized that bCBT is equally as effective as regular face- to-face CBT (fCBT), but that intervention costs for blended CBT will be reduced.

## Methods/design

### Study design

**Fig. 1:**
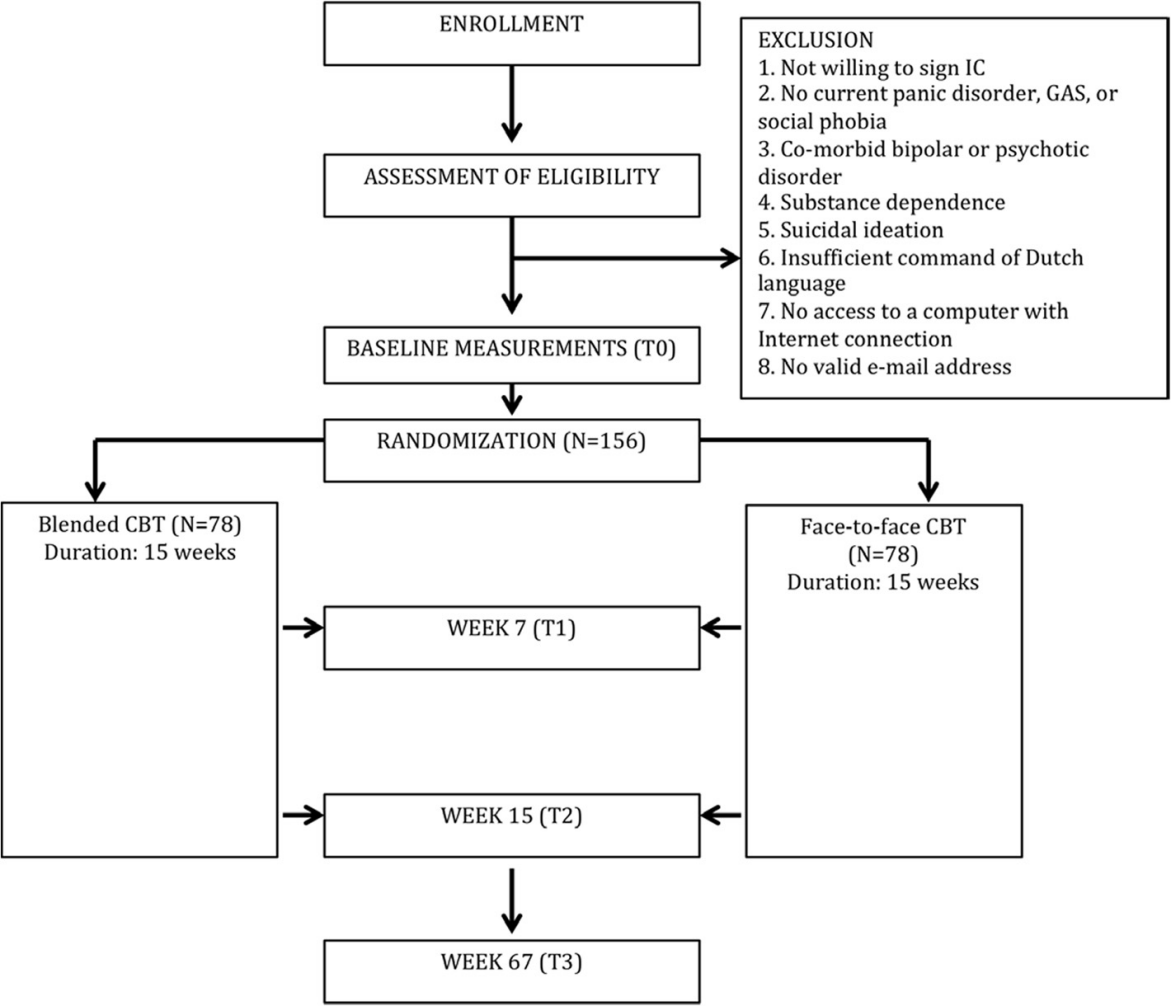
Flowchart of the study design

The study is designed as a parallel-group randomized controlled equivalence trial (*N* = 156), in which patients with panic disorder, social phobia or generalized anxiety disorder are randomly allocated to either bCBT (*N* = 78) or fCBT (*N* = 78). The protocol for this study has been approved by the Medical Ethics Committee of the VU University Medical Centre, Amsterdam (registration number 2015.073). Written informed consent will be obtained from all participants. Figure 1 displays the flowchart of the study design, in accordance with the CONSORT guidelines [28, 29].

### Measurements

Measurements are taken at baseline (T0) and at three fixed intervals after the first treatment session; at week 7 (T1), 15 (T2) and 67 (T3). Questionnaires are self-administered online. The diagnostic interviews will be administered by a trained researcher face-to-face at the mental health care location. Appendix: Table 1 provides an overview of the measures that are used at each time point.

### Participants

#### Inclusion criteria

Patients aged 18 years and older are eligible to participate if they meet the criteria for a DSM-V diagnosis of a severe anxiety disorder (social phobia, panic disorder with or without agoraphobia and generalized anxiety disorder). The Structured Clinical Interview for DSM disorders axis I (SCID-I) [30] or the MINI international Neuropsychiatric Interview plus (MINI plus) [31, 32] will be performed face-to-face by a trained researcher to assess these inclusion criteria.

#### Exclusion criteria

Patients are excluded from the study if they i) do not have adequate proficiency in the Dutch language, both verbal and written, ii) do not have a valid e-mail address and a computer with Internet access, iii) suffer from one or more of the following disorders: a psychotic disorder, bipolar disorder and/or substance dependence, iv) are identified to be at high risk for suicide. The SCID-I [30] or the MINI plus [31, 32] will be used to assess whether the exclusion criteria iii) and iv) apply. Comorbid disorders other than psychotic and bipolar disorders are allowed, as is psychopharmacological treatment.

Excluded participants will be offered one of the regular treatment options within the participating specialized mental health care centre. For respondents with a heightened suicide risk, the principal investigator will inform the professional responsible for treatment immediately via telephone and e-mail.

### Recruitment

Patients will be recruited at the anxiety disorder departments of three large scale specialized mental health care centres in the Netherlands. All newly registered patients undergo an intake interview by an experienced clinician, after which diagnosis and treatment is established and discussed with the patient.

Subsequently, eligible patients are informed about the study by the researcher. Interested patients will then receive an information brochure and an informed consent form via e-mail and will be invited to take the baseline diagnostic interview. During this interview, a trained researcher will confirm the primary diagnosis of panic disorder, social phobia or generalized anxiety disorder and assess comorbidity. If patients are willing and eligible to participate, written informed consent will be requested.

### Randomization and blinding

Participants will be randomly assigned to either bCBT or fCBT by an independent researcher, based on a computer-generated block randomization table [28].

Randomization will be stratified by research site to control for the differences between centres. Group allocation cannot be blinded to patients and therapists because they will obviously notice whether they perform or receive bCBT or fCBT.

### Interventions

#### Blended cognitive behavioural therapy

bCBT is a protocolized manualized treatment consisting of 15 sessions, with weekly alternating 45-min face-to-face sessions and online sessions (approximately 50-50 %) with online feedback from the therapist. Online sessions are accessible in a secure web-based environment (Minddistrict; www.minddistrict.com). Patients and therapists access this platform with a personalized login. Performing an online session will take patients approximately 45 min, and providing online feedback will take therapists approximately 15 min per patient per session. Feedback messages are sent on the online platform to ensure secure communication.

The blended protocol is based on evidence-based protocols for treatment of anxiety disorders and recommendations in national and international treatment guidelines [8–10]. The protocol was developed in collaboration with patients, therapists and experts through organized focus groups during the development phase of the blended intervention, who provided feedback on the content and presentation.

Key elements of bCBT are psycho-education (explanation of the treatment rationale and the general procedures in cognitive therapy), cognitive therapy (examining relationships between thoughts, emotions and behaviour), interoceptive exposure and exposure in vivo (exposure to feared situations) and relapse prevention (identifying and adopting strategies to prevent anxiety symptoms from re-occuring). After a face-to-face introduction session with an explanation of bCBT, the treatment starts with a face-to-face session and it also ends with a face-to-face session.

The online sessions have a fixed structure that starts with therapy information, followed by multiple exercises and homework assignments. The sessions contain text boxes with information and testimonials from fictional patients and videos in which a therapist explains the theory. Patients get online feedback from their therapist on finished exercises at a fixed day and time. Homework assignments are discussed in the subsequent face-to-face session.

On completion of treatment, patients can continue to access the online treatment platform in order to reread information and look up homework exercises, such as the relapse prevention plan.

#### Face-to-face cognitive behavioural therapy

The fCBT entails fifteen weekly 45-min face-to-face sessions with psycho-education, cognitive therapy, interoceptive exposure, exposure in vivo and relapse prevention. Therapists follow a protocol with the same content as the bCBT protocol.

### Therapists

All participating therapists are experienced clinicians and will be trained in the bCBT protocol and the fCBT protocol prior to the study. During the training they are informed about the content and the structure of the protocol and they receive instructions about how to work with the online platform. Therapists work with both treatment groups. During the trial, therapists will attend peer group supervision meetings every other week. The supervision meetings are guided by the head researcher at the centre (an experienced psychologist) and the research coordinator.

### Clinical outcome measures

#### Severity of anxiety symptoms

The Beck Anxiety Inventory (BAI) [33] will be used to measure the severity of anxiety symptoms at every assessment moment (T0-T3). The BAI is a reliable and well-validated measure of somatic anxiety symptoms found across the anxiety disorders [34]. It consists of twenty-one questions about how the subject has been feeling in the last week, expressed as common symptoms of anxiety (such as numbness and tingling, sweating not due to heat, and fear of the worst happening). Each question has the same set of four possible answer options, which are arranged in columns and are answered by marking the appropriate one with an X. The BAI has a maximum score of 63. For this study, treatment response is defined as a symptom reduction of the baseline BAI symptom severity score of at least 30 % and remission a score reduction of at least 30 % plus a total score <11, based on validation in The Netherlands Study of Depression and Anxiety (NESDA) [35–37].

### Measures of quality adjusted life years

#### General well-being

The EQ-5D-5L [38, 39] will be administered at all time points (T0-T3) to assess health related quality of life. This validated questionnaire consists of five questions that tap mobility, self-care, daily activities, pain and mood. Each item has five response categories. The labels for each of the dimensions are: no problems, slight problems, moderate problems, severe problems and incapacity/extreme problems. In addition to this, participants use a VAS scale to rate their health on a scale ranging from 0 (*worst possible health*) to 100 (*best possible health*). The answers to the five questions are combined in a number sequence that corresponds with the five answers. Each sequence corresponds to a certain health state. On these health states, a value (utility) has been placed [40], which in turn is used to determine the quality-adjusted life years (QALYs). To obtain a utility score per patient, the area-under-the curve method (AUC) will be applied [41]. This method consists of linearly interpolating between the different health states at the different time points. Subsequently, the area under the curve is calculated.

### Cost calculations

The cost-effectiveness will be assessed taking a societal and health care perspective. Cost within health care, costs to the patient and productivity costs are taken into account. The Treatment Inventory of Costs in Psychiatric patients (TiC-P) will be applied to collect input data on costs. The TiC-P is a validated comprehensive questionnaire focused on establishing costs incurred within and outside the health care system as well as costs due to productivity losses [42].

#### Health care utilization costs and patient costs

Part 1 of the TiC-P is a validated instrument that measures the direct medical costs by calculating the number of contacts with health care services (general practitioner, psychiatrist, medical specialist, physiotherapist, alternative health practitioner, day care/hospital length of stay), during the last three months. Also, information about the number of contacts and time spent by the patient on the online part of the intervention will be collected. Additionally, patients’ out-of-pocket costs, such as the costs of travelling to the health services and the patients’ time costs of travelling are determined.

Apart from these costs, the costs of offering the treatments will be taken into account. For example the costs of developing and maintaining the online part of the treatment, as well as the costs of weekly therapist online feedback. The costs are calculated by multiplying the volumes by the corresponding reference unit prices [43].

#### Productivity costs

The second part of the TiC-P contains the iPCQ. This part asks questions about productivity losses that are caused by absence (absenteeism), reduced efficiency at work (presenteeism) and difficulties in job performance. Sickness absence for less than one month is defined as short-term absence, and sickness absence for more than one month as long-term absence. If respondents indicated that they had been absent for the entire recall period, data were collected from the time when the period of long-term absence started. This additional information will be used to value the production losses according to the friction cost method [44]. This method takes into account the economic circumstances that limit the losses of productivity to society, which are related to the fact that a formerly unemployed person may replace a person who becomes disabled. Productivity losses were valued according to the average value added per worker by age and gender per day and per hour prices [43].

### Other variables of interest

To further evaluate bCBT compared to fCBT, a number of explorative measures are administered.

#### General patient characteristics and treatment preference

Demographic characteristics such as age, sex, education, employment and marital status will be collected with a general demographic questionnaire at baseline (T0). Additional questions are asked concerning clinical anxiety characteristics such as age of onset, number of months with an anxiety disorder in past 4 years, duration of current episode, somatic illnesses and treatment status. In addition, participants are asked about their computer use: number of hours spent at a computer and reasons for use. Finally, patients indicate their treatment preference (bCBT/fCBT).

#### Depression

The Beck Depression Inventory-II (BDI-II) [45] is a 21-question multiple choice self-report inventory of the most widely used instruments for measuring the severity of depression and assesses the presence and severity of depressive symptoms. The BSI-II has been validated in Dutch [46]. It will be used at every time point (T0-T3).

#### Work and social adjustment

The Work and Social Adjustment Scale (WSAS) [47] is a 5-item patient self-report measure, which assesses the impact of a person’s mental health difficulties on their ability to function in terms of work, home management, social leisure, private leisure and personal or family relationships at all time points (T0-T3). The WSAS is used for all patients with depression or anxiety as well as phobic disorders. It is a reliable and valid measure [47].

#### General psychopathology

The Brief Symptom Inventory (BSI) [48] is a 53-item, self-report symptom inventory designed to evaluate general psychopathology at every time point (T0-T3). It is a brief form of the SCL-90 and is designed to provide a multidimensional symptom measurement in about 10 min. The questionnaire has been validated in Dutch [49].

#### Locus of control

The five-item version of The Mastery Scale [50] is administered at each assessment moment (T0-T3) to assess changes in *locus of control*. Locus of control could potentially mediate treatment effect and facilitate relapse prevention. The questionnaire consists of five questions, which are scored on a five-point Likert-scale, ranging from 1 (*totally disagree*) to 5 (*totally agree*). The total score ranges from 5 to 30, with higher scores being indicative of a higher level of experienced control. The scale has good psychometric properties [50].

#### Therapeutic alliance

The Revised Short Version of the Work Alliance Inventory (WAI-SR) [51, 52] is used to let patients rate the *quality of the work alliance* between patient and therapist at T1 (week 10). The questionnaire is administered to investigate whether the blended treatment has an effect on the quality of the work alliance.

The questionnaire consists of 12 items, which are scored on a five-point Likert-scale, ranging from 1 (seldom or never) to 5 (always). The raw scores range from 12 to 60, with higher scores being indicative of a better alliance between therapist and patient. The questionnaire has satisfactory psychometric properties [51].

#### Treatment evaluation

The Client Satisfaction Questionnaire-8 (CSQ-8) [53] will be administered at week 15 (T2). The CSQ consists of 8 questions with item-specific response categories. The total score ranges from 8 to 32, with higher scores being indicative of higher *levels of client satisfaction*. The CSQ-8 has a high internal consistency [54].

The System Usability Scale (SUS) [55] will be administered at week 15 (T2) amongst the participants randomized to the bCBT group. The SUS consists of 10 questions with 5 response options, ranging from 0 (strongly disagree) to 4 (strongly agree). The total scores are converted to a scale ranging from 0 to 100. Higher scores are indicative of higher usability of the online platform that is used for bCBT. It has been found to be a reliable questionnaire [56].

#### Process data

Data for process analyses are obtained from the administration of the participating mental health care institutions and through usage statistics of the online platform. We will consider the following aspects:

- Recruitment: time required for the recruitment of patients
- Treatment adherence: percentage of dropout during therapy, number of completed sessions, reasons for treatment dropout, number of face-to-face contacts and number of cancellations, homework adherence
- Time investment: by both the patient and the therapist

### Sample size

In economic evaluations we are calculating the power to estimate the joint distribution of costs and treatment effects. Subsequently, we need more information for estimating power compared to clinical trials, namely expected costs of treatments, expected covariance of treatment effects/costs, and the maximum willingness to pay for the treatment effect. To incorporate this information, the formula of Glick et al. [57] can be used. A goal of sample size and power calculation for cost-effectiveness analysis is to identify the likelihood that an experiment will allow us to be confident that a therapy is acceptable or not when we adopt a particular willingness to pay.

For this study a sample size of 156 is based on a formula to estimate the power of a cost-effectiveness analyses.

$$ n=\frac{2{\left({z}_a+{z}_{\beta}\right)}^2\left(s{d}^2+\left({W}^2s{d}^2\right)-\left(2W\rho s{d}_cs{d}_q\right)\right)}{{\left( WE-C\right)}^2} $$

_Where:_

N = sample size/group

*z*_α_ = z statistic for alpha

*z*_β_ = z statistic for beta

sdc = Expected standard deviation costs

sde = Expected standard deviation effects

W = Willingness to pay

C = Expected differences in costs

E = Expected differences in effects

ρ = correlation between differences in costs and effects

(*z*_α_ = 1,96; *z*_β_ = 0,84; sdc = 800; sde = 0,02; W = 80,000; C = 832; E = 0,02; ρ = 0,1)

Based on the literature of the similar effectiveness of iCBT compared to fCBT, we will conduct an equivalence study to show that bCBT and fCBT do not differ significantly in their short- and long-term effectiveness (expected between-groups effect-size *d* of 0.2). The sample size in this equivalence study is based on an applied equivalence limit difference ES of 0.4, as this range of small to moderate difference in effect size will not result in clinically important differences. The power of this study that both treatments are similar is set at 0.80 with an alpha of 0.05 to calculate sample size and resulted in the inclusion of 78 patients per condition (total *n* = 156). This was supported by the estimates based on the formula.

### Statistical analysis

#### Primary analysis

A cost-effectiveness analysis (CEA) will be conducted from the societal perspective. In addition, a budget impact analysis (BIA) will be based on a health-economic modelling study in accordance with Mauskopf’s recommendations [58], i.e. from the perspective of the health care decision maker.

#### Cost-effectiveness analysis

Costs will be assessed at pre-, post-treatment and at one-year follow up. As the TIC-P cost date covers a period of three months, all costs will be extrapolated to a 12 month period, assuming stability of costs during the time frame. A multilevel model (to correct for correlation between measurements) with a link function (as cost-data will not be normally distributed) is used to obtain parameter estimates, likelihood and p-values for the costs and effects. The fitted estimates will be bootstrapped to assess confidence intervals [59]. An incremental cost-effectiveness ratio (costs per case response or remission) will be calculated (ICER = (mean costs bCBT treatment-mean costs fCBT)/(mean bCBT − mean fCBT). The mean costs, including all costs, of the patients in the bCBT condition will be subtracted from the mean costs of the patients in the fCBT condition. This difference will then be divided by the subtracted effects (case of response or remission on the BAI) and an estimation of the bCBT treatment groups’ incremental costs in relation to their incremental health benefit will be generated. Additionally, an incremental cost-utility ratio (costs per QALY) will be calculated; this procedure is identical to the cost-effectiveness ratio with the exception that instead of the cost per QALY, the cost per case of response or remission, is calculated. Finally, to test the robustness of the results, we will conduct sensitivity analyses, to investigate how sensitive the ICERs will be to changes of cost estimates (for example difference in costs per bCBT contact, type of psychologists and number of sessions). For decision-making purposes, the ICER acceptability curve will be plotted for various willingness-to-pay (WTP) ceilings, which helps in making judgments about whether the blended intervention offers good value for money, relative to treatment as usual. One-way sensitivity analyses directed at uncertainty in the main cost drivers will be performed to gauge the robustness of our findings across a range of likely values for those parameters.

### Budget impact analysis

To assess how health care budgets are changed by offering bCBT for anxiety compared to fCBT, a *budget impact analysis* (BIA) will be conducted as outlined in Mauskopf et al. [58]. The BIA will include 1) the perspective of the public purse (in Dutch: Budgettair Kader Zorg), and 2) the perspective of the health care decision makers. We consider costs when 10, 20, 30 and 100 % of the target group receive bCBT compared to fCBT. These scenarios will be compared with the base-case scenario, reflecting current care, where 0 % of the target group is offered blended CBT. The BIA will be conducted taking account of the perspectives of health care decision makers. For this, the average remuneration rates of the Dutch Health Authority will be used (NZa). The Budget Impact Analysis (BIA) will be conducted using a health economic (Markov cohort) simulation model.

### Explorative analyses

Outcomes on continuous clinical outcome variables, such anxiety symptoms, at T1, T2 and T3 (week 7, 15, and 67) are estimated for descriptive purposes through mixed-model analyses (MM), with participants as random effects, and time (T1-T3), group (blended vs. face-to-face treatment) and time x group as fixed effects, with baseline scores as a single covariate. Missing data will be imputed statistically. To assess the magnitude of treatment effects, Cohen’s *d* effect sizes [60] for each time point are calculated by dividing MM parameter estimates of fixed effects at each post-treatment assessment by the pooled standard deviation of outcome measurements at baseline (T0).

## Discussion

The study described is a randomized controlled trial in which the health care efficiency of bCBT for adults with panic disorder, social phobia or generalized anxiety disorder in outpatient specialized mental health care is examined. The main goal is to assess the cost-effectiveness of bCBT in comparison to fCBT, from a societal and a health care perspective.

Both national and international studies have shown that the costs of anxiety disorders are substantial. This is reflected in health care costs and loss of productivity. bCBT has the potential to increase the cost-effectiveness compared to fCBT, mainly due to its effectiveness combined with less therapist time needed and fewer patients’ visits to therapist. bCBT may also increase patients’ self-management; they have more control over time and frequency of treatment, because they can access the online platform as often and as long they want, in combination with therapist support. The fact that blended CBT may benefit patients and therapists and can be executed quite easily and possibly at less cost than conventional CBT, means that it is potentially very interesting for health care institutions to be able to deliver this type of treatment, and for health care insurance companies to include these treatments in their reimbursement programs.

However, clinical and economical evaluations of this type of treatment are still scarce. Several studies confirm the effectiveness and cost-effectiveness of iCBT for depression and anxiety disorders [23], but none of these studies investigated cost-effectiveness of blended CBT for anxiety disorders in specialized mental health care. By adopting a societal perspective in this study all relevant information that may be of interest for the decision-making process is incorporated in the analysis. Hence, in this study, patients’ time and productivity costs are part of the assessment.

A strong feature of the current trial is that therapy content of fCBT and bCBT is similar, captured in a protocol for both conditions. Both interventions entail clinical behavioural therapy and exposure, a daily routine treatment for anxiety disorders. In addition, the recruitment of patients and inclusion and exclusion criteria are similar to the usual procedures in mental health organizations, which enhances the external validity of the results that will be obtained.

The strength of high external validity is simultaneously a limitation with regard to internal validity. The study is designed to closely adhere to established procedures in routine practice in outpatient specialized mental health care, which can make it difficult to attribute clinical results to the blended treatment. However, with this study we want to gain insight into the cost-effectiveness of bCBT, rather than its clinical effectiveness.

Furthermore, we aim to collect follow-up data after a year. Therefore, an inherent challenge to the study is retention. To minimize drop-out, reminders for filling in questionnaires will be sent by e-mail and if deemed necessary, participants will be called personally to remind them and possibly fill in the questionnaire together during the phone call. To handle missing data, we will impute missing values statistically.

## Appendix

**Table 1:** Overview of measures administered at each assessment interval

Questionnaire	Aim	Baseline (T0)	Week 7 (T1)	Week 15 (T2)	Week 67 (T3)
Primary outcomes					
BAI	*Anxiety severity*	x	x	x	x
SCID-I/MINI-plus	*Diagnostic interview*	x			
EQ-5D-5L	*General well-being*	x	x	x	x
TiC-P	*Health-care utilization*	x		x	x
Other variables of interest					
General patient characteristics		x			
A priori treatment preference		x			
BDI	*Depression*	x	x	x	x
WSAS	*Work and social adjustment*	x	x	x	x
BSI	*General psychopathology*	x	x	x	x
CSQ	*Satisfaction*			x	
Mastery Scale	*Locus of control*	x	x	x	x
WAI	*Therapeutic alliance*		x		
SUS (bCBT only)	*System usability*			x	

*BAI* Beck Anxiety Inventory, *SCID* Structured Clinical Interview for DMS disorders axis I, *MINI plus* Mini International Neuropsychiatric Interview Plus, *EQ-5D-5L* EuroQol, *iC-P* Trimbos and iMTA questionnaire on Costs associated with Psychiatric illness, *BDI* Beck Depression Inventory, *WSAS* Work and Social Adjustment Scale, *BSI* Brief Symptom Inventory, *CSQ* Client Satisfaction Questionnaire, *WAI* Work Alliance Inventory, *SUS* System Usability Scale, *bCBT* blended Cognitive Behavioural Therapy
